# Beyond real: alternative unitary cluster Jastrow models for molecular electronic structure calculations on near-term quantum computers

**DOI:** 10.1039/d5sc03585f

**Published:** 2025-11-05

**Authors:** Nikolay V. Tkachenko, Hang Ren, Wendy M. Billings, Rebecca Tomann, K. Birgitta Whaley, Martin Head-Gordon

**Affiliations:** a Department of Chemistry, University of California Berkeley CA 94720 USA nikolay.tkachenko@ou.edu whaley@berkeley.edu m_headgordon@berkeley.edu; b Materials Sciences Division, Lawrence Berkeley National Laboratory Berkeley CA 94720 USA; c Department of Chemistry and Biochemistry, University of Oklahoma Norman Oklahoma 73019 USA; d Institute for Decarbonization Materials, University of California Berkeley California 94720 USA; e Chemical Sciences Division, Lawrence Berkeley National Laboratory Berkeley CA 94720 USA

## Abstract

Near-term quantum devices require wavefunction ansätze that are expressive while also of shallow circuit depth in order to both accurately and efficiently simulate molecular electronic structure. While the unitary coupled cluster ansatz (*e.g.*, UCCSD) has become a standard, the high gate count associated with the implementation of this limits its feasibility on noisy intermediate-scale quantum (NISQ) hardware. *k*-Fold unitary cluster Jastrow (uCJ) ansätze mitigate this challenge by providing O(*kN*^2^) circuit scaling and favorable linear depth circuit implementation. Previous work has focused on the real orbitalrotation (Re-uCJ) variant of uCJ, which allows an exact (Trotter-free) implementation. Here we extend and generalize the *k*-fold uCJ framework by introducing two new variants, Im-uCJ and g-uCJ, which incorporate imaginary and fully complex orbital rotation operators, respectively. Similar to Re-uCJ, both of the new variants achieve quadratic gate-count scaling. Our results focus on the simplest *k* = 1 model, and show that the uCJ models frequently maintain energy errors within chemical accuracy (∼1 kcal mol^−1^). Both g-uCJ and Im-uCJ are more expressive in terms of capturing electron correlation and are also more accurate than the earlier Re-uCJ ansatz. We further show that Im-uCJ and g-uCJ circuits can also be implemented exactly, without any Trotter decomposition. Numerical tests using *k* = 1 on H_2_, H_3_^+^, Be_2_, C_2_H_4_, C_2_H_6_ and C_6_H_6_ in various basis sets confirm the practical feasibility of these shallow Jastrow-based ansätze for applications on near-term quantum hardware.

## Abstract 2

Im-uCJ and g-uCJ ansätze extend orbital rotations into complex space. Implemented exactly *via* generalized Givens rotations, these ansätze achieve higher accuracy and more robust optimization than the currently available Re-uCJ.

## Graphical abstract

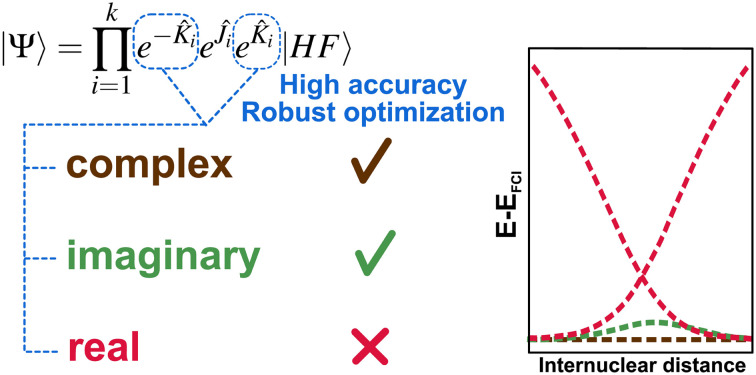

## 1 Introduction

Electronic structure simulations represent one of the most promising scientific application areas where quantum computers have the potential to outperform classical methods. Since *ab initio* calculations play an essential role in various fields from catalysis to drug discovery, the significant advantages that quantum computers are expected to provide relative to classical approaches would open new possibilities for the prediction and analysis of complex systems.^1,2^ In molecules lacking strong static electron correlations, methods such as Density Functional Theory (DFT)^3,4^ or wave function-based techniques such as coupled cluster (CC) methods^5–9^ often produce usefully accurate results, and have been successfully employed for decades. Nevertheless, both approaches have inherent drawbacks. On the one hand, the accuracy of DFT typically depends on the functional chosen, as well as the system under investigation, and can further be constrained by self-interaction errors. On the other hand, the most precise wave function-based methods are computationally expensive, limiting their use to small or moderately sized systems. Furthermore, in the case of strongly correlated systems, despite their accuracy, even the most advanced classical algorithms based on complete active space (CAS) methods,^10–13^ the density matrix renormalization group (DMRG),^14–16^ or selected configuration interaction^17^ face unfavorable exponential scaling with molecule size (measured, *e.g.*, based on the number of atoms, electrons, and spatial or spin orbitals), thereby restricting their application to small systems.

A wide variety of algorithms have been proposed for efficient electronic structure calculations on quantum computers.^1,2,18–20^ While the quantum phase estimation (QPE) algorithm^21^ offers a scalable pathway for addressing the electronic structure problem,^22^ the high circuit costs of this algorithm and its variants^23^ appear to require fault-tolerant quantum hardware.^24–26^ The majority of algorithms proposed for the simulation of electronic structure on near-term noisy, intermediate scale quantum (NISQ) devices fall either within the category of variational optimization algorithms^27–31^ or lie within the broad class of quantum subspace diagonalization (QSD)^32–35^ algorithms. In the variational optimization category, the variational quantum eigensolver (VQE)^27^ is perhaps the most well-known. VQE uses a quantum computer to prepare trial wavefunctions and a classical computer to optimize the parameters of these wavefunctions. While initial studies indicated significant promise for ground-state energy calculations,^27,28^ subsequent work showed that the classical optimization component is non-trivial, due to the frequent occurrence of barren plateaux that arise from shallow or even flat energy landscapes.^36–39^ Several extensions and modifications of VQE have since been developed to improve its accuracy and applicability.^40–49^ For example, PermVQE^47^ introduces correlation-informed qubit permutation to the optimization process, allowing the accuracy of energy predictions to be improved without increasing the circuit depth. Another example is ADAPT-VQE,^48^ which optimizes construction of the quantum circuit by selectively adding operators that provide the greatest reduction in energy at each iteration. The more recent qubit-ADAPT-VQE^49^ further enhances the performance of this adaptive approach by optimizing the operator selection at the qubit-operator level, thereby reducing the number of quantum gates required for efficient simulations.

An alternative class of quantum algorithms for calculating electronic energies on NISQ or near-term hardware is provided by subspace diagonalization algorithms. These focus on capturing electron correlation by expanding the state basis. They are hybrid algorithms that generally involve classical postprocessing of a Hamiltonian eigenvalue problem with the matrix elements evaluated on a quantum processor. These algorithms can be further classified according to the methods used to define the basis states, which may or may not be orthogonal. For instance, the Quantum Subspace Expansion (QSE) builds on VQE outputs by constructing an expanded subspace from the original VQE solution, *e.g.*, *â*^†^_p_*â*_q_|*Ψ*_VQE_〉.^50,51^ It is also possible to achieve provable convergence with respect to growth of the subspace when the set of expanded states forms a Krylov basis that is generated by the repeated application of the matrix of interest to an initial guess vector.^52^ Several algorithms have been proposed along this direction, including quantum filter diagonalization,^33^ quantum Lanczos,^53^ and quantum Davidson methods.^54^ A different form of subspace diagonalization is provided by the non-orthogonal quantum eigensolver (NOQE),^55^ which is a multi-reference method for systems with both strong and weak electronic correlations that offers both algorithmic and practical quantum advantages, and has recently been shown to provide a complexity theoretic quantum speedup for such systems.^56^

The work we report here focuses on the adaptations needed to successfully use cluster wavefunctions as reference states for systems with both strong and weak electronic correlations. As such, it is relevant to both variational methods and the NOQE. Classical CC theory converges only slowly with rank for strongly correlated systems^57^ due to both its nonvariational character and the nature of the cluster expansion.^58^ The variational issue is well solved *via* the VQE and unitary CC (UCC) theory.^27,59–63^ However, the large number of variational parameters, even at the lowest singles and doubles (*i.e.*, quartic scaling amplitudes at the UCCSD level) which results in large circuit depths has motivated development of more compact alternatives.^40,64,65^ We focus here on the unitary cluster Jastrow approximation (uCJ),^66^ which builds on the well-known real-space Jastrow factors from quantum Monte Carlo, extended into Hilbert space to become cluster operators.^67,68^ The uCJ approach was used in the NOQE^55^ where the reduction in gate count was noted, and has also been successfully mapped onto aspects of both physics of strong correlation and qubit connectivity with a local uCJ extension.^69,70^

In this work, we introduce and systematically explore several variants of the uCJ ansätze, analyzing their circuit depth, expressibility, and accuracy by use of calculations that minimize the Hamiltonian energy for individual ansatz states. Specifically, we examine two new forms of the uCJ correlator, one with imaginary orbital rotation operators (Im-uCJ) and one with complex orbitalrotation operators (g-uCJ). We show that similar to the previously introduced Re-uCJ ansatz,^55^ these new ansätze also reduce the gate count relative to the widely used UCCSD ansatz, making the uCJ correlators more suitable for near-term hardware. We demonstrate that both Im-uCJ and g-uCJ can achieve high accuracy, often surpassing both UCCSD and their real-rotation counterpart (Re-uCJ), while preserving a shallow circuit depth. We benchmark these uCJ ansätze on a series of small molecules, demonstrating that cluster Jastrow correlators can serve as a key step toward practical and resource-efficient quantum algorithms for molecular electronic structure on NISQ and near-term devices.

## 2 Theory

### 2.1 Formulations of unitary cluster Jastrow ansätze

While UCC ansätze, such as UCCSD, are frequently used in variational quantum algorithms, their circuit depths often become impractical on near-term quantum hardware. One path toward lowering this overhead is to move away from two-body operators and build wavefunction ansätze from simpler one-body terms. In this work, we therefore focus on Jastrow-style correlators, which use exponentials of one-electron operators and particle number operators.^66^ The *k*-fold uCJ ansatz is expressed as1
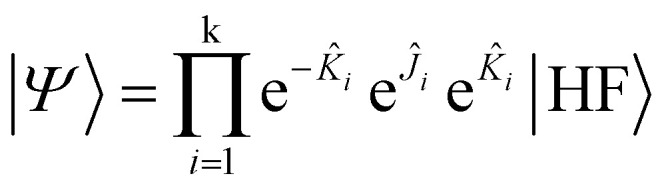
where the operators *K̂* and *Ĵ* are defined as2
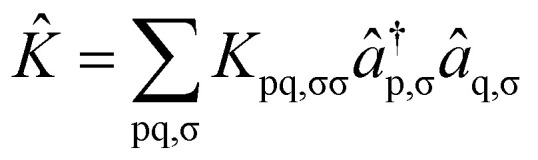
3
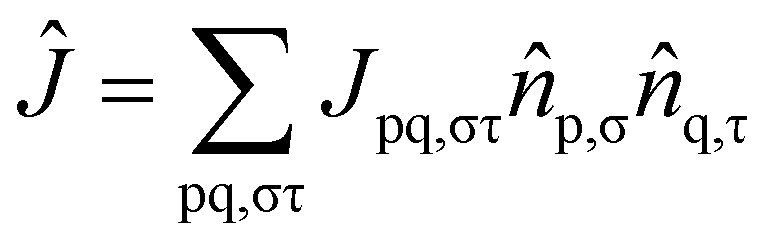
In the present formulation of the *K̂* operator, we restrict orbital rotations to occur only within the same spin subspace. This choice constrains the number of variational parameters and simplifies the ansatz. However, lifting this restriction (allowing rotations connecting different spin states) increases the number of variational parameters (while still scaling quadratically with the number of spin orbitals) and can enhance expressibility. In this work, we primarily investigate the spin-preserving form, and will state explicitly when the spin-generalized form of *K̂* is used.

In eqn (1)–(3), p and q refer to spatial molecular orbitals, σ and τ represent spin polarization (either *α* or *β*), and |HF〉 is the mean-field restricted or unrestricted Hartree–Fock reference state. The parameter *k* controls how many replicas of the Jastrow type correlators are included in the ansatz. If *k* is not truncated, eqn (1) can be exact.^59,66^ Nevertheless, we shall limit ourselves to *k* = 1 since this choice is the simplest and cleanest, and we wish also to explore the extent to which this can provide a good balance between capturing a significant portion of electron correlations while maintaining a shallow enough ansatz to be interesting for near-term devices. For notational simplicity, we will omit the σ subscripts in *K*_pq,σσ_, while noting that the orbital rotations only couple orbitals of the same spin. To maintain unitarity in the uCJ ansätz, the coefficients *K*_pq_ and *J*_pq,στ_ must satisfy specific conditions: the matrix **K** is required to be anti-Hermitian, while **J** must be purely imaginary and symmetric.^66^

Previous studies^55,66,69^ have explored the uCJ ansatz with real orbital rotations, which imposes the restriction that **K** is real and anti-Hermitian. We refer to this form as real uCJ (Re-uCJ). In this case, the effective form of the *K̂* operator can be written as:4
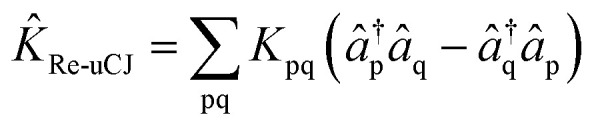


However, the flexibility of the uCJ ansatz also allows additional choices that to our knowledge have not yet been explored. It is particularly interesting to explore whether different choices can yield significant improvements for truncation at *k* = 1 that we impose throughout this work.

The first alternative we consider is the use of the *K̂* operator with the opposite restriction to that imposed in Re-uCJ, *i.e.*, restricting **K** to be imaginary. We thereby obtain another form of the uCJ ansatz, which we shall refer to as imaginary uCJ (Im-uCJ):5
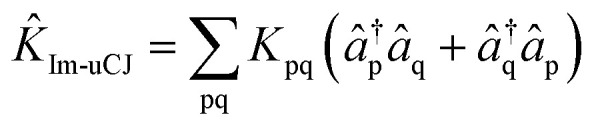


The second alternative is to consider the least restricted scenario for **K**, where it is allowed to be complex with both real and imaginary parts. This formulation provides the greatest flexibility for a given truncation of *k*, and therefore we refer to it as generalized uCJ (g-uCJ). The *K̂* operator for g-uCJ is written as:6
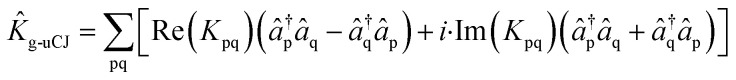


The overall scalability of the Jastrow ansatz is significantly more favorable than other UCC variants, since the number of terms in e^*K̂*_*i*_^ and e^*Ĵ*_*i*_^ scale as O(*N*^2^), where *N* is the number of spin orbitals. This results in an overall O(*N*^2^) scaling of the uCJ ansatz, in contrast to the formal O(*N*^4^) scaling for a single Trotter step of a UCC ansatz with double excitations.^71^ Additionally, by applying a fermionic-to-spin transformation to *K̂* (*e.g.*, the Jordan–Wigner (JW) transformation^2,72^), we find that the restricted Re-uCJ and Im-uCJ ansätze are both represented with a number of Pauli words that is smaller by a factor of two than that for the g-uCJ ansatz. Once the ansatz is defined, the parameters in the matrices **K** and **J** can be optimized either (i) purely classically, for example through methods such as classical variational Monte-Carlo,^67,68^ or (ii) within the VQE framework. In this work, we test the performance of all three uCJ ansatz variants, Re-uCJ, Im-uCJ, and g-uCJ.

### 2.2 Implementation of the Im-uCJ and g-uCJ ansätze through Givens rotations

Upon transforming the operators *K̂* and *Ĵ* from the fermionic to a qubit representation, the question arises of how to accurately represent the exponentials of these operators. In general, once *K̂* and *Ĵ* are mapped into qubit space, they may consist of Pauli words that do not commute with each other. A straightforward solution to this non-commutativity is to employ the approximate Trotter decomposition. However, for the specific case of the JW transformation, the need for Trotter decomposition can be avoided. In the JW mapping, the exponentiation of the *Ĵ* operator is straightforward to handle, since the number operators are mapped to commuting *Ẑ* Pauli operators, according to7
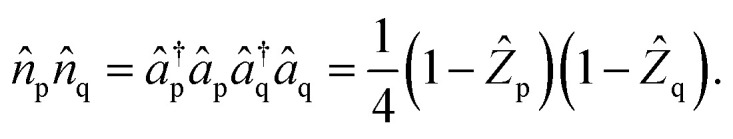


The JW mapping then allows us to express the sum of these operators as an exact product of exponentials of individual Pauli terms.

On the other hand, the JW transformed *K̂* operator generally includes non-commuting terms. Implementation of this operator presents a greater challenge, but can be realized by taking advantage of the demonstration that any real unitary orbital rotation operator can be implemented efficiently using Givens rotation operators.^73^ Here we extend this approach to show that the exponentials exp(*K̂*_Im-uCJ_) and exp(*K̂*_g-uCJ_) can also be represented as consecutive applications of a generalized form of Givens rotation.

The analysis in ref. 73 showed that any particle number-preserving rotation operator of the single-particle basis8
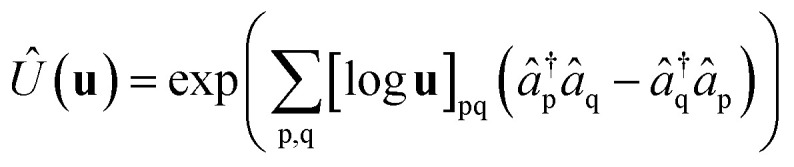
where **u** is a unitary matrix, can be efficiently decomposed into a sequence of 
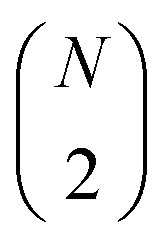
 real fermionic rotations of the form

9 *R̂*_pq_(*θ*_k_) = exp(*θ*_k_(*â*^†^_p_*â*_q_ − *â*^†^_q_*â*_p_)),

The proof is based on the equivalence of application of the orbital rotation operator *R̂*_pq_(*θ*_k_) to the unitary *Û*(**u**) and rotations of matrix **u**

10 *R̂*_pq_(*θ*_k_)*Û*(**u**) = *Û*(**r**_pq_(*θ*_k_)**u**),

where **r**_pq_(*θ*_k_) denotes a Givens rotation, represented by an *N* by *N* matrix (with *N* as the number of spin–orbitals considered) of the form11
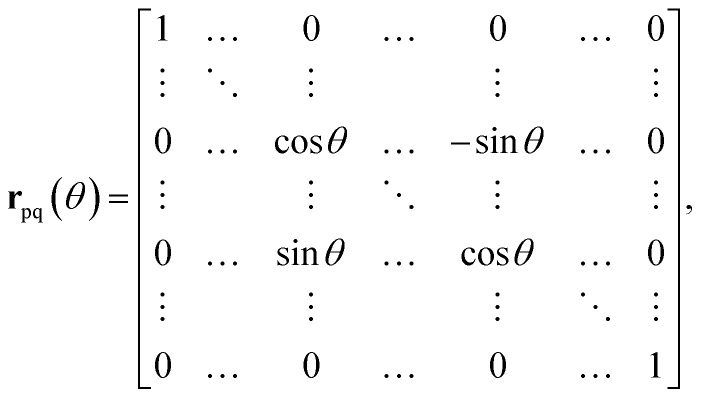
where the cosine terms occupy the (p,p) and (q,q) positions and oppositely signed sine terms occupy the (p,q) and (q,p) positions. By identifying a sequence of Givens rotations **r**_pq_(*θ*_k_) that diagonalize the matrix **u**, which can be done by a QR-like decomposition as described in ref. 73, one has12
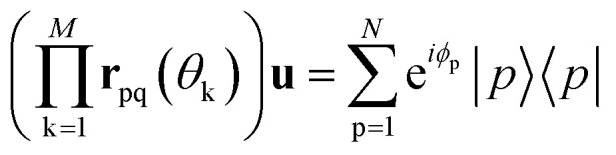
and substituting this entire sequence in eqn (10) leads to13
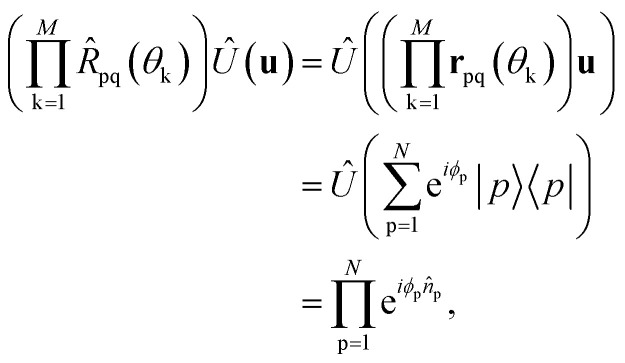
from which the implementation of *Û*(**u**) follows by applying the one-qubit phase gates 
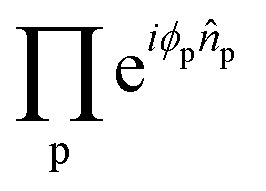
, followed by the inverse of the sequence of two-qubit rotations *R̂*_pq_(−*θ*_k_):14
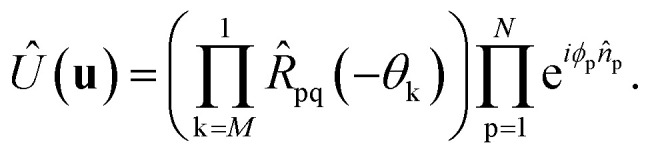


This implementation can be directly applied to the Re-uCJ ansatz by choosing the unitary operator **u** as

15 **u** = exp(**K**),

so that the unitary *Û*(**u**) in eqn (8) and (14) correspond to the real e^*K̂*^ operator, which thus represents a real orbital rotation unitary and can be then implemented by using eqn (14). However, the decomposition in ref. 73 does not directly apply to the Im-uCJ and g-uCJ ansätze, which involve imaginary and complex orbital rotations, respectively, and therefore the corresponding matrices **u** are no longer real. To extend the method to be applicable with complex **u**, we use a generalized Givens rotation of the form16
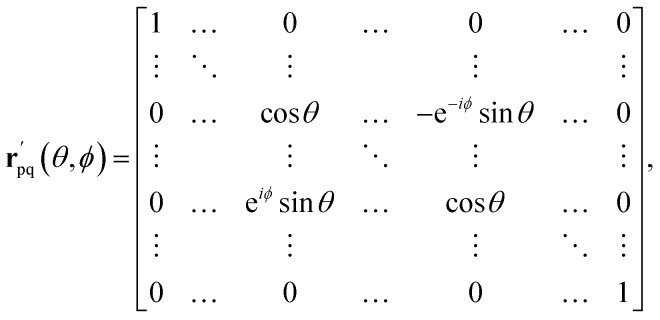
together with the corresponding generalized fermionic rotations17
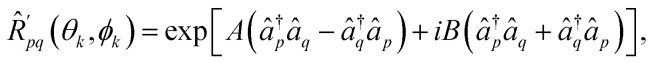
where the angles *θ* and *ϕ* are defined as 
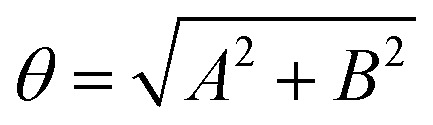
 and 
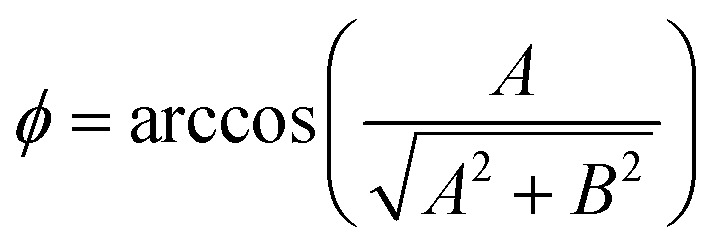
. With these generalized rotation operators in hand, we can apply the same procedure derived in ref. 73 and summarized above, but replacing *R̂*_pq_ by 
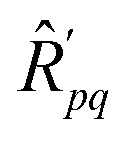
, and **r**_pq_(*θ*) by 
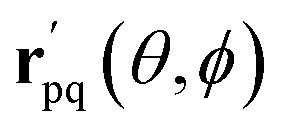
. The values *θ* = *θ*_k_ and *ϕ* = *ϕ*_k_ for the complex-valued rotation matrix 
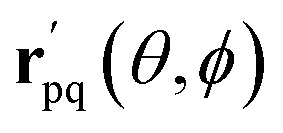
 that diagonalizes the corresponding complex-valued matrix **u** (*cf.*eqn (12)) are obtained from a corresponding generalization of the QR-like decomposition that is given explicitly in the SI. As a result, the general complex orbital rotation matrix for the Im-uCJ and g-uCJ ansatz can be decomposed into 
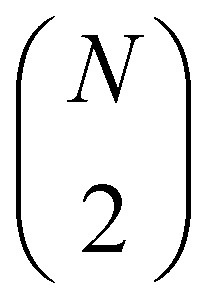
 generalized fermionic rotation operators, according to18
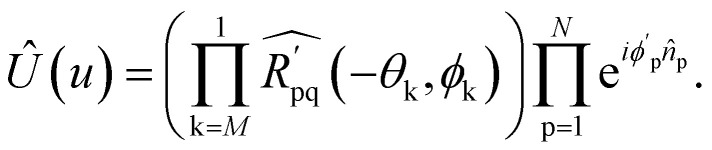


By further restricting the fermionic rotations to adjacent qubits we avoid non-local lengthy JW *Ẑ* Pauli strings and use only local Givens rotations operators. With this realization, the operator 
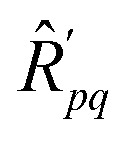
 is effectively a two-qubit operator, which can be realized with the use of only three CNOT gates.^74–76^

One of the important benefits of using exact exponentiation over Trotter decomposition is the fact that we can then effectively treat biradicaloid systems when using the NOQE.^55^ In a typical biradicaloid, pairs of unrestricted Hartree–Fock reference states differ only by permutations of spin orbitals, allowing the same **K** and **J** matrices to be reused for each reference simply by permuting the corresponding matrix indices. This reuse significantly reduces the cost of parameter optimization, as only one set of parameters must be optimized. The energy accuracy can then be improved by incorporating additional references with no more classical preprocessing costs, although there is now the additional quantum processor expense to measure more nondiagonal overlap and Hamiltonian matrix elements. By contrast, in an approximate Trotter decomposition scheme, these parameters must generally be re-optimized for each reference state, removing the classical preprocessing efficiency that exact exponentiation affords.

### 2.3 Explicit illustration of ansatz construction with H_2_ STO-3G

To illustrate the distinctions between the three uCJ ansätze, we consider an illustrative example of a four spin–orbital, two-electron system: the H_2_ molecule in the STO-3G basis set. Given spin-orbitals *ϕ*^σ^_*i*_ (*σ* = *α*, *β*) and using the Jordan–Wigner mapping with the convention that occupied orbitals are listed first, we represent our wavefunction in the occupation vector form as follows: |*ϕ*^*α*^_1_*ϕ*^*β*^_2_*ϕ*^*α*^_3_*ϕ*^*β*^_4〉_, where orbitals *ϕ*^α^_1_*ϕ*^β^_2_ are occupied and *ϕ*^α^_3_*ϕ*^β^_4_ are virtual spin–orbitals in the HF state. The explicit form of the operator *K̂*_g-uCJ_ is:

19 *K̂*_g-uCJ_ = *K*_13_*â*^†^_1_*a*_3_ + *K*_24_*â*^†^_2_*a*_4_ + *K*_31_*â*^†^_3_*a*_1_ + *K*_42_*â*^†^_4_*a*_2_.

Since **K** is anti-Hermitian, we have20
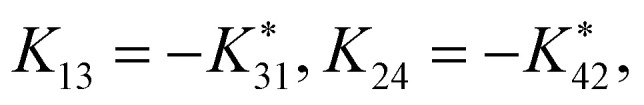
which translates to

21 Re(*K*_13_) = −Re(*K*_31_), Re(*K*_24_) = −Re(*K*_42_), Im(*K*_13_) = Im(*K*_31_), Im(*K*_24_) = Im(*K*_42_).

Now, let us express the *K̂* operator in terms of Pauli words using the Jordan–Wigner transformation:22
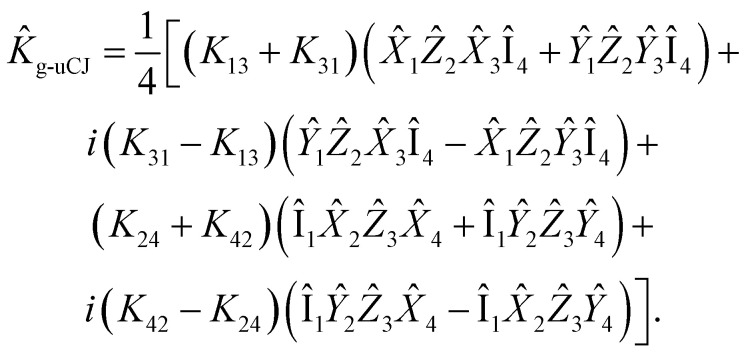


Given the anti-Hermitian nature of the **K** matrix, the expression above can be rewritten as:23
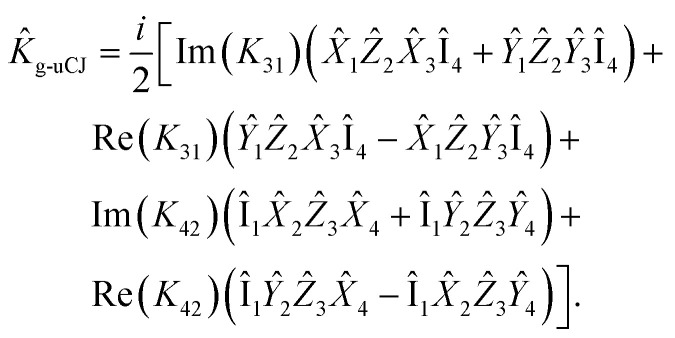


Thus, there are four real parameters to optimize for this operator. However, by restricting Re(*K*_*ij*_) = 0 (Im-uCJ, eqn (5)) or Im(*K*_*ij*_) = 0 (Re-uCJ, eqn (4)), we reduce the number of parameters to two:24
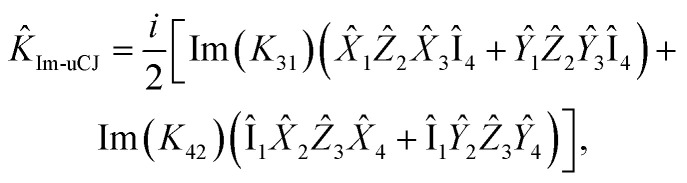
25
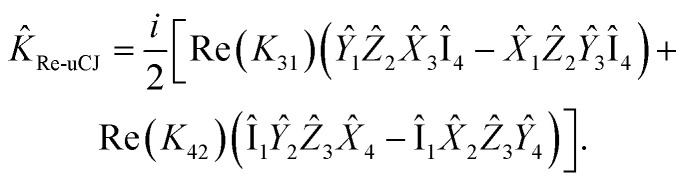


Since [*n̂*_*i*_, *n̂*_*j*_] = [*â*^†^_*i*_*â*_*i*_, *â*^†^_*j*_*â*_*j*_] = 0, and given that **J** is symmetric and purely imaginary, the explicit form of the *Ĵ* operator for our two electron, four spin–orbital singlet case is:

26 *Ĵ* = 2[*J*_12_*â*^†^_1_*â*_1_*â*^†^_2_*â*_2_ + *J*_14_*â*^†^_1_*â*_1_*â*^†^_4_*â*_4_ + *J*_23_*â*^†^_2_*â*_2_*â*^†^_3_*â*_3_ + *J*_34_*â*^†^_3_*â*_3_*â*^†^_4_*â*_4_ ]

Using the JW transformation, we can show that:27
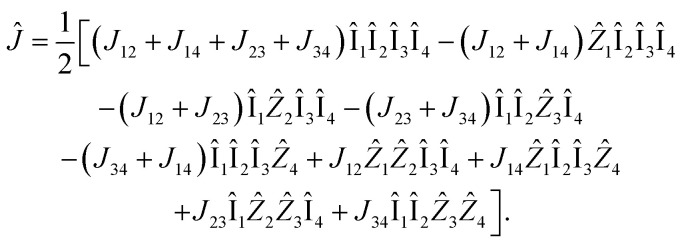


This yields four variational parameters to optimize. Note that we assumed *n̂*_1_*n̂*_3_|*Ψ*〉 = *n̂*_2_*n̂*_4_|*Ψ*〉 = 0, given the singlet multiplicity of the wavefunction for ground state H_2_. In the general case, without this assumption, there would be six variational parameters to optimize for **J**.

### 2.4 Technical details

We have examined several chemical systems, H_2_, H_3_^+^, Be_2_, C_2_H_4_, C_2_H_6_ and C_6_H_6_, to evaluate and compare the performance of the VQE algorithm with each of the three uCJ ansätze. The RHF solutions were generated using the PySCF software package.^77^ The exponentiation of the *K̂* operator in the Re-, Im-, and g-uCJ circuits was implemented exactly through the Givens rotation as described in Section 2.2 above. For the C_2_H_6_, Be_2_, C_2_H_4_, and C_6_H_6_ molecules, the CASCI formalism was used with active spaces of (2e,2o),(2e,2o),(4e,4o), and (4e,4o), respectively (we denote the number of spatial orbitals o here, according to quantum chemistry convention, and use *N* to denote the number of spin orbitals, according to quantum computing convention). The corresponding active space orbitals are illustrated in Fig. S2. For benzene, we employed a (4e,4o) active space focusing on the frontier HOMO–LUMO, since the fully bonding π and fully antibonding π* orbitals are energetically distant and contribute minimally to the correlation energy. CASCI calculations confirm that expanding from (4e,4o) to a (6e,6o) active space changes the total correlation energy by only ≈2%. Corresponding energies for both (4e,4o) and (6e,6o) active spaces are provided in the SI file (Table S2).

To analyze the gate counts for the circuits, we performed UCCSD simulations performed with the Qiskit-nature package.^78,79^ For the single Trotter-step decomposed ansätze (UCCSD, Re-uCJ, Im-uCJ, and g-uCJ), the number of CNOT gates was computed by first performing the Jordan–Wigner transformation of the *T̂*_1_, *T̂*_2_, *Ĵ* and *K̂* operators, followed by simplifying the resulting Pauli word sums through term cancellation, and finally representing each remaining exponentiated Pauli word by efficient quantum circuits as described in ref. 80. For exact implementations of the uCJ ansätze, the exponential e^*Ĵ*^ was similarly decomposed by obtaining its JW representation, recognizing that in this case all Pauli words contain only *Z* and *I* gates and thus pairwise commute, allowing the exact decomposition into a product of exponentials of individual Pauli words. The e^*K̂*^ and e^−*K̂*^ terms were implemented by applying 
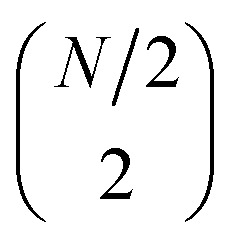
 generalized orbital rotation operators 
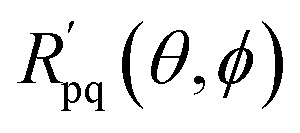
 as described in Section 2.2, where *N* represents the total number of spin orbitals, and division by two accounts for spin–orbital rotations only within the same spin space.

Optimization of the **K** and **J** matrices was carried out classically with an implementation of the SLSQP minimizer,^81^ using a sparse-vector representation of quantum states. The optimization minimizes the expectation value of the Hamiltonian in each of the reference states, by generating 〈*Ĥ*〉 using uCJ states from **K** and **J** matrices and variationally optimizing the expectation value with respect to their parameters. This classical optimization represents a pre-processing stage that is not susceptible to the measurement shot noise or circuit noise endemic to quantum processors. For larger active spaces (greater than or equal to 8 qubits), optimization of the **K** and **J** matrices was first performed in a reduced space under the perfect pairing assumption (only one bonding and its corresponding antibonding orbital are entangled at a time through the **K** and **J** operators). The orbital connectivity was then gradually extended toward the fully connected case, using the optimized coefficients from the previous step as the initial guess for each subsequent stage. The sparse-vector simulator implementation was written locally, and can be accessed through GitHub.^82^

In order to assess the performance of the ansätze in a realistic setting on current NISQ hardware, we undertook circuit-based simulations. Two types of these simulations were performed. The first type, run on the QASM simulator, includes only shot noise from measurement sampling and was used to assess the robustness of a variational optimization on a quantum device. The Jordan–Wigner Hamiltonian was partitioned according to the qubit-wise commuting (QWC) approach^83^ (Table S3) where the Pauli strings are grouped into sets in which each Pauli string commutes with every other string in a qubit-wise manner. This allows all terms in a group to be sampled simultaneously. Each group can then be measured by making appropriate one-qubit rotations then measuring in the computational basis. We used 10 000 shots per group for optimization in this setting without circuit noise. Parameters were optimized with the Powell optimizer.^84^ The final energy evaluations with optimized parameters were repeated for over 20 independent trials using 10 000 shots per QWC group.

The second type of simulation includes both the shot noise and realistic circuit noise (including readout noise). The Qiskit Aer backend was used here to introduce hardware noise models. For these simulations we evaluated the energy expectation value on optimized uCJ ansatz states for which the **K** and **J** parameters were taken from the noiseless classical optimizations described above. The circuits were executed on the same backend for four hardware noise models. We denote these as (i) a “*T*_1_/*T*_2_″ model incorporating only decoherence noise, (ii) a “*T*_1_/*T*_2_ + dep” model that adds light single- and two-qubit depolarizing noise, (iii) a “H1-like” model that resembles the Quantinuum H1 device with asymmetric readout errors using parameters from ref. 85, and (iv) an “IBM-like” model that resembles the IBM Pittsburgh device using parameters from ref. 86. For detailed description of these noise models see Table S4 in the SI. For these simulations with circuit noise, each QWC group was evaluated with 20 000 shots and repeated over 100 independent trials with different random seeds. The IBM-like circuits (for an “IBM-like” model) were compiled into the native gate basis *C*_Z_, *R*_ZZ_, *R*_X_, *R*_Z_, *S*_*x*_, *X*, while the H1-like circuits (for “*T*_1_/*T*_2_”, “*T*_1_/*T*_2_ + dep”, and “H1-like” models) used *R*_Z_, *R*_X_, *R*_ZZ_ gates, corresponding to the physical single-qubit and entangling operations of the corresponding hardware platforms. All-to-all qubit connectivity was assumed in these noisy simulations. The test was conducted for H_2_ in the STO-3G basis at *R*(H–H) = 1.7 Å, (a point on PES where the energy difference between ansätze is the most pronounced).

## 3 Results for molecular benchmarks

### 3.1 Circuit depth analysis

We begin our discussion with an analysis of circuit depth for the investigated ansätze. As shown in Table 1, the exact implementations of the three uCJ ansatz variants result in a substantially smaller number of native two-qubit gates (CNOTs) compared to UCCSD circuits. As expected, this difference becomes more pronounced for larger systems. For example, for the 12-qubit H_2_ system in the 6-311G basis, the exact implementations of all uCJ variants require approximately six times fewer two-qubit gates than the single-step Trotter-Suzuki decomposition of UCCSD (192 *vs.* 1202 two-qubit gates). We note that this reduction is not observed when using single-step Trotter-Suzuki decompositions of the uCJ ansätze. In fact, Table 1 shows that in this situation, the g-uCJ variant can in some cases perform worse than UCCSD. Therefore, we recommend using exact implementations of the uCJ ansätze whenever possible.

**Table 1:** Comparison of the number of two-qubit gates for the systems considered in this manuscript. *N*_2q_ stands for the number of native two-qubit gates (CNOTs) in the circuit, “single T-step” refers to the Trotter–Suzuki decomposition with one step, and “exact” refers to a uCJ implementation that avoids Trotter-Suzuki decomposition as discussed in Section 2.2

System	*N*_elec_	*N*_qubit_	UCCSD *N*_2q_	g-uCJ *N*_2q_		Re-uCJ *N*_2q_		Im-uCJ *N*_2q_	
			Single T-step	Single T-step	Exact	Single T-step	Exact	Single T-step	Exact
H_2_ (STO-3G)	2	4	42	46	20	36	20	34	20
Be_2_ (STO-3G, (2e, 2o))	2	4	42	46	20	36	20	34	20
C_2_H_6_ (STO-3G, (2e, 2o))	2	4	42	46	20	36	20	34	20
H_3_^+^ (STO-3G)	2	6	166	166	54	120	54	126	54
H_2_ (6-31G)	2	8	404	376	104	264	104	270	104
H_4_ (STO-3G)	4	8	753	400	128	288	128	294	128
C_2_H_4_ (STO-3G, (4e, 4o))	4	8	753	400	128	288	128	294	128
C_6_H_6_ (STO-3G, (4e,4o))	4	8	753	400	128	288	128	294	128
H_2_ (6-311G)	2	12	1202	1319	192	894	192	902	192

We now compare the performance of the restricted *versus* general uCJ ansätze. For single-step Trotterized circuits, the restricted Re-uCJ and Im-uCJ variants of uCJ employ approximately two-thirds the number of two-qubit gates compared to the generalized g-uCJ ansätz. This behavior arises from the two-fold increase in the number of Pauli terms in the definition of the *K̂* operator for g-uCJ (see eqn (23)–(25)). In contrast, for the exact implementations of Im-uCJ and g-uCJ introduced in this work that use the generalized Givens rotations, the CNOT cost is similar across all three uCJ variants in their exact form (see eqn (12)–(16)), although the number of variational parameters differs between restricted and generalized variants.

As we demonstrate in the Performance analysis section below, the increased number of variational parameters in g-uCJ is offset by its greater flexibility and expressivity. In all of the considered cases, the g-uCJ ansatz recovers a larger portion of the correlation energy than its restricted counterparts and often yields results within chemical accuracy. If the number of variational parameters is a critical constraint, we recommend using the restricted variants (Re-uCJ or Im-uCJ), with the remaining correlation energy recovered using, for example, the NOQE algorithm.^55^

### 3.2 Performance analysis

**Fig. 1:**
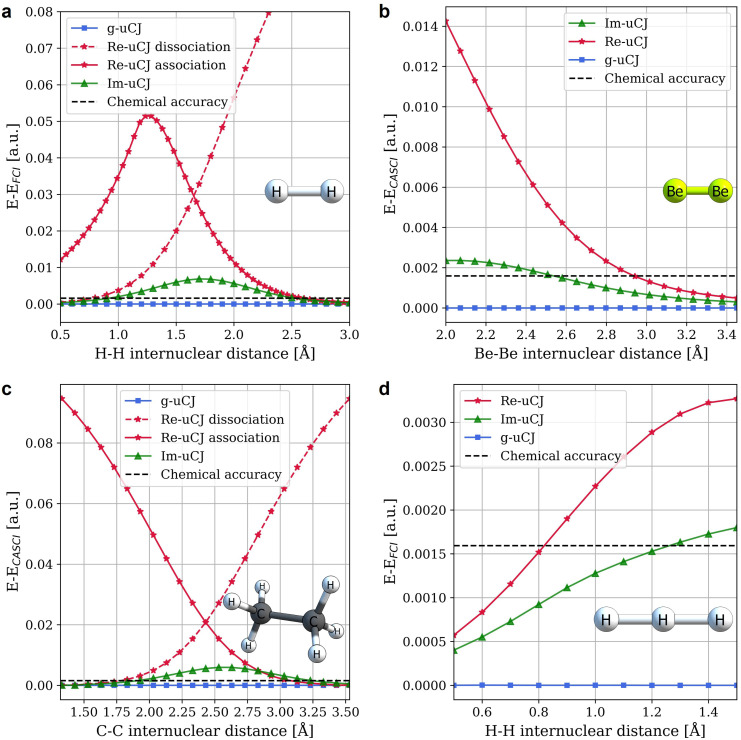
Errors in energies for variational single reference calculations with g-uCJ, Re-uCJ and Im-uCJ ansätze relative to FCI or CASCI energies for (a) H_2_, (b) Be_2_ (2e,2o), (c) C_2_H_6_ (2e,2o), and (d) linear H_3_+. The STO-3G basis set was used for all molecules. The horizontal black dashed line corresponds to chemical accuracy (∼1 kcal mol^−1^, equivalent to 1.6 × 10^−3^ a.u.). The RHF state was used as a reference state for all systems. The curves denoted by ‘association’ or ‘dissociation’ differ in how the initial guesses for the **K** and **J** matrices were obtained. For the association curves, the optimized parameters from the prior step were used as the initial guess at the next step as the bond distance was gradually decreased. In contrast, for the dissociation curves, the bond distance was gradually increased.

We begin our performance analysis with the H_2_ molecule in the STO-3G basis set (Fig. 1a). Bond dissociation curves were calculated to evaluate the performance of the three uCJ ansätze across different correlation regimes. We considered an RHF reference for all molecules, but for H_2_ we also compared results obtained from UHF reference states. Beyond the Coulson–Fischer point, a single-reference ansatz based on UHF becomes a symmetry-broken wavefunction and the resulting energies show greater error than the ansätze built on RHF references (see Fig. S1 in the SI). We also see that for all except very small internuclear distances, the energies obtained with single-reference Im-uCJ are systematically lower than those obtained with single-reference Re-uCJ. Notably, the g-uCJ ansatz exhibits sufficient flexibility to exactly reproduce the FCI energies for H_2_ in STO-3G, a trend that holds across all the two-electron problems examined in this work.

As illustrated for H_2_, Be_2_ (2e,2o), C_2_H_6_ (2e,2o), and linear H_3_^+^ in Fig. 1, the performances of the Im-uCJ and Re-uCJ ansätze show significant differences. Despite requiring the same number of two-qubit gates, Im-uCJ consistently yields more accurate results, whereas Re-uCJ sometimes converges to metastable local minima and struggles with optimization. This behavior is clearly illustrated in Fig. 1a and c. Here the Re-uCJ curves denoted as ‘dissociation’ were obtained by gradually increasing the bond distance and using the optimized coefficients from each prior step as an initial guess. Due to the lack of flexibility of the Re-uCJ ansatz, this approach accumulates errors as the bond breaks, as is also seen and well-known for RHF ansätze. The curves denoted as ‘association’ were generated by gradually decreasing the bond distance, with each calculation initialized using the optimized coefficients from the previous step. In the bond breaking regime, the energy still can be reduced by variational optimisation of the Re-uCJ parameters, but the results remain inferior to Im-uCJ (except for the C_2_H_6_ case with *R*_CC_ > 2.9 Å, where Re-uCJ performs slightly better than Im-uCJ), and this variational local minimum becomes inferior to Re-uCJ ‘dissociation’ results at shorter bond distances (*R*_HH_ < 1.6 Å for H_2_, and *R*_CC_ < 2.4 Å for C_2_H_6_). We note that random initialization of the Re-uCJ parameters along the dissociation curve leads to erratic optimization behavior due to jumping between the dissociation and association curves, disrupting smoothness of the energy as a result. In contrast, Im-uCJ reliably converges to a single lower energy solution regardless of the initial guess procedure, demonstrating greater stability.

Similar results are observed for the other molecular systems shown in Fig. 1b and c, although there are some specific features worth commenting on. In particular, we see that Be_2_ exhibits no binding in calculations using an RHF reference, while in fact it is known from experiments to have a weak bond, with *R*_e_ = 2.45 Å. Part of its strong correlation effect can be captured with a (2e,2o) active space, allowing 
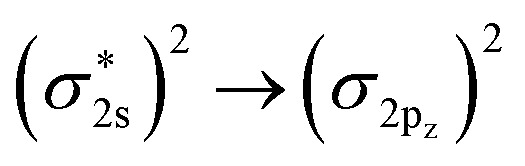
 excitations, as illustrated in Fig. S2b. The electron correlations are stronger at *R*_e_ than at dissociation, as reflected in the sharp rise in the Re-uCJ error at short distances in Fig. 1b. Similar to the dissociation of H_2_, for Be_2_ we see that Im-uCJ again provides dramatic improvement over Re-uCJ, while g-uCJ attains exactness at all distances.

To further assess the robustness of the proposed ansätze in practice, we carried out simulations that introduce both measurement shot noise as well as quantum hardware noise. First, Fig. S3 presents the PES of H_2_ computed with the three uCJ variants using the QASM simulator and including only statistical shot noise. The simulations show that the Im-uCJ and g-uCJ ansätze retained their higher accuracy relative to Re-uCJ, indicating that they not only achieve better energies but also retain stable optimization behavior under sampling fluctuations.

Second, to quantify the impact of quantum device noise derived from both physical gate and measurement noise sources, Table 2 reports the results of hardware-noise simulations performed for the same H_2_ system at an internuclear distance of 1.7 Å (the point of maximum energy deviations among the three ansätze on the PES). For this test, we used the classically optimized parameter sets and introduced representative noise models, including ones that correspond to Quantinuum H1-like and IBM-like devices (see Section 2.2 and the SI for more details). We find that across all models tested here, the relative accuracy of the ansätze remains unchanged. Specifically, g-uCJ consistently exhibits the smallest deviation from the FCI reference, while Re-uCJ exhibits the largest error. These results demonstrate that the qualitative performance order of the uCJ variants is also preserved under realistic hardware noise conditions. It is important to note, however, that even for the g-uCJ ansatz, the realistic gate, readout, and decoherence error rates (H1-like and IBM-like models) on current quantum devices (without any form of noise mitigation and error correction) produce energy deviations of approximately 1.4 × 10^−2^–2.6 × 10^−2^ a.u. relative to the exact FCI solution, as shown in Table 2. While both of these lie outside chemical accuracy (∼1 kcal mol^−1^, equivalent to 1.6 × 10^−3^ a.u.), the results with circuit noise emulating ion-trap platforms such as Quantinuum H1 exhibit smaller deviations, due to their higher single- and two-qubit gate fidelities. These results illustrate the limitations of NISQ era hardware, which are expected to recede as we move into the pre-fault-tolerant era where use of error-detecting codes with post-selection, additional layers of error mitigation, and eventually also some error correction, will significantly improve the performance.

**Table 2:** Energies, standard deviations (*σ*), and errors in energy relative to the FCI energy (Δ*E*), for the three uCJ variants under different noise models for H_2_ in the STO-3G basis at 1.7 Å internuclear distance. Noise model ‘none’ denotes no circuit noise but inclusion of measurement shot noise. The other noise models also include circuit noise, with variable components (see Section 2.4 and the SI for more details on the noise model definitions and corresponding simulations). The FCI reference energy is *E*_FCI_ = −0.9714267 a.u

Ansatz	Noise model	*E*_mean_ (a.u.)	*σ* (a.u.)	Δ*E* (a.u.)
Re-uCJ	None	−0.938640	0.001052	0.032786
	*T*_1_/*T*_2_	−0.932796	0.001091	0.038630
	*T*_1_/*T*_2_ + dep	−0.932253	0.001111	0.039173
	H1-like	−0.926495	0.001152	0.044931
	IBM-like	−0.916108	0.001205	0.055319
Im-uCJ	None	−0.964422	0.001057	0.007004
	*T*_1_/*T*_2_	−0.957332	0.001089	0.014095
	*T*_1_/*T*_2_ + dep	−0.956925	0.001110	0.014501
	H1-like	−0.950271	0.001130	0.021155
	IBM-like	−0.938314	0.001258	0.033112
g-uCJ	None	−0.971274	0.001126	0.000152
	*T*_1_/*T*_2_	−0.964271	0.001137	0.007146
	*T*_1_/*T*_2_ + dep	−0.963883	0.001102	0.007544
	H1-like	−0.957142	0.001131	0.014285
	IBM-like	−0.945106	0.001271	0.026319

**Fig. 2:**
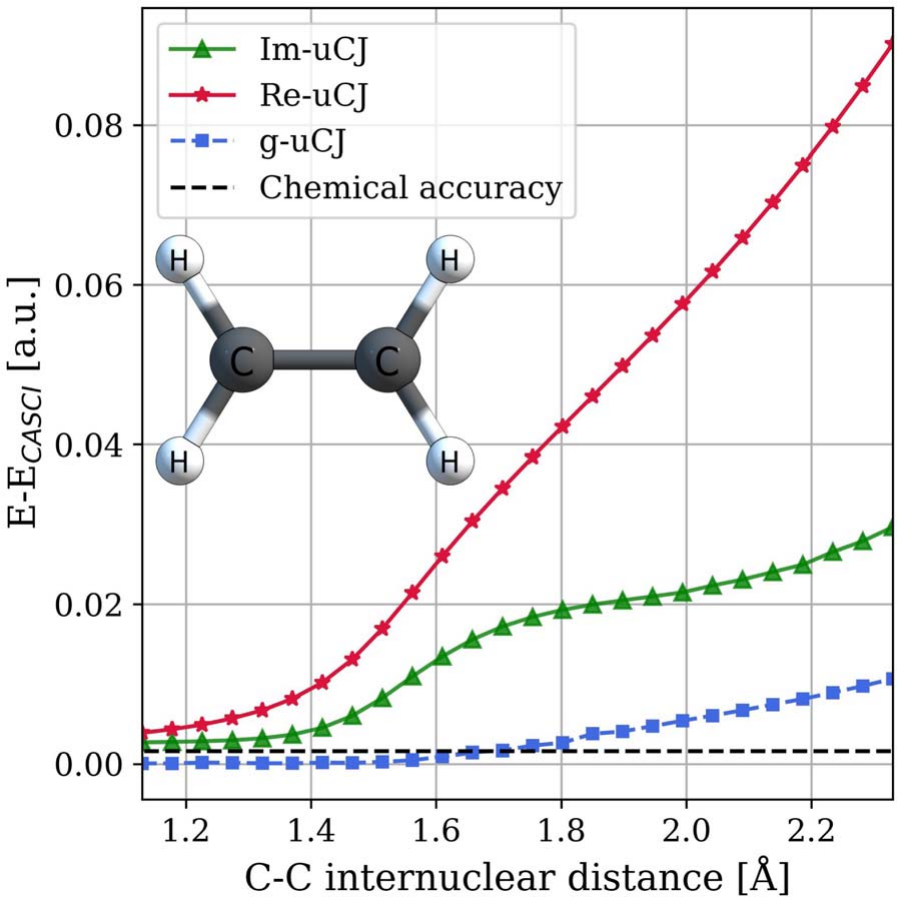
Errors in energies for g-uCJ, Re-uCJ and Im-uCJ relative to CASSI energies for C_2_H_4_ (4e,4o) at the STO-3G basis set. The horizontal black dashed line corresponds to chemical accuracy (1.6 mHa, equivalent to 1.6 × 10^−3^ a.u.). The RHF state was used as a reference state.

While exactness for two-electron systems is a desirable property and can be achieved for H_2_ by optimization of both g-uCJ and UCCSD ansätze, realistic applications typically involve larger numbers of electrons in the active space. To assess the performance of the uCJ variants in such settings, we examined the case of a 4-electron, 4-orbital system corresponding to the double bond breaking in C_2_H_4_. The results are shown in Fig. 2. As can be seen here, the same general trend persists, namely that g-uCJ performs best, while Re-uCJ yields the least accurate results. Notably, g-uCJ maintains chemical accuracy up to a C–C bond distance of 1.7 Å. At larger separations, its performance degrades due to the inability to fully describe the dissociation into two ^3^CH_2_ radicals, a process that involves quadruple excitations, which requires going beyond the *k* = 1 level of a uCJ ansatz. Thus, in a situation where bonds with double or higher order bond character are broken at larger distances, it is recommended to go beyond the *k* = 1 level of the uCJ ansatz.

For larger systems, single-point energy calculations were performed using the g-uCJ, Re-uCJ, and Im-uCJ ansätze. These are summarized in Table 3, where the previously observed trends are maintained. Thus, Re-uCJ generally recovers the smallest fraction of the correlation energy, Im-uCJ performs better, and of the three ansätze, g-uCJ captures the largest fraction of the correlation energy. A comparison with UCCSD is also provided in Table 3. As shown there, g-uCJ achieves accuracy comparable to UCCSD while requiring significantly fewer two-qubit gates. Since symmetry breaking can contribute to the correlation energy in some systems, we also report the percentage of correlation energy captured by a single broken-symmetry UHF reference state, without dressing by a cluster Jastrow operator (last column of Table 3). As shown in line 2 of Table 3 for the highly correlated H_4_ molecule in a square geometry, using a bare UHF reference state captures approximately ≈87% of the electronic correlation energy, while applying the g-uCJ ansatz to the RHF reference improves the accuracy, recovering around ≈95% of the correlation energy and bringing the result significantly closer to the FCI energy. To further improve upon the accuracy achieved with g-uCJ, algorithms such as NOQE and, in the long term, QPE can be employed.

**Table 3:** Percentage of the total correlation energy (relative to FCI or CASCI solutions) that is recovered with g-uCJ, Re-uCJ, and Im-uCJ ansätze for three different molecular systems

System	UCCSD	g-uCJ	Re-uCJ	Im-uCJ	UHF
H_2_ (6-31G, *R*_HH_ = 1.2 Å)	100	100	82.88	99.96	0.06
H_4_ (STO-3G, *R*_HH_ = 1.1 Å)	92.84	94.56	89.76	92.01	87.02
C_6_H_6_ (STO-3G, (4e,4o))	100	92.26	59.74	83.97	N/A

We note that for even larger systems, the VQE optimization of uCJ circuits is expected to pose challenges such as barren plateaus, sensitivity to initialization and convergence difficulties. Empirically, we observe that Im-uCJ is less sensitive to initial conditions than Re-uCJ, suggesting that imaginary orbital rotations provide additional flexibility that stabilizes convergence. Similar observations have been made in recent studies, where initializing generalized gates in complex space was found to improve optimization performance, even when the exact ground state is real.^87,88^ This suggests that access to complex rotations provides additional flexibility that reduces the susceptibility to fall into local minima.

### 3.3 Configuration state function analysis

**Fig. 3:**
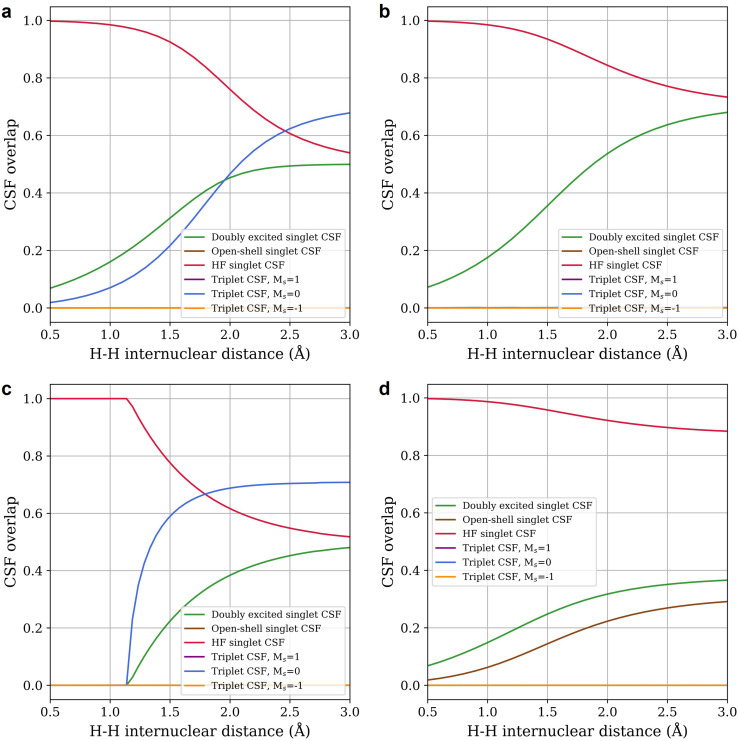
Overlaps of the configuration state functions (CSFs) with the wavefunction obtained after application of (a) Im-uCJ, (b) g-uCJ, (c) Re-uCJ association, and (d) Re-uCJ dissociation ansätze for the H_2_ molecule in the STO-3G basis set. The RHF state was used as a reference state for all systems. The association and dissociation curves for a given ansatz differ in how the initial guesses for the **K** and **J** matrices were obtained. For association (dissociation) curves, the optimized parameters from the prior step were used as the initial guess at the next step as the bond distance was gradually decreased (increased) (see the text).

To better understand why Im-uCJ yields more accurate energies than Re-uCJ, we performed a configuration state function (CSF, spin-adapted linear combination of determinants) composition analysis of the corresponding wavefunctions for H_2_. The results are summarized in Fig. 3. The exact FCI ground state wavefunction should include only two singlet CSFs: the Hartree–Fock state and the doubly excited singlet state, both of gerade (g) symmetry. This expected behavior is observed for the g-uCJ ansatz in Fig. 3b, where only these two CSFs contribute to the wavefunction, leading to a solution that is correct in both energy and spatial and spin symmetry.

Interestingly, we find that the Im-uCJ ansatz exhibits a different structure. As shown in Fig. 3a, in addition to the HF and doubly excited CSFs, the wavefunction also includes contributions from the triplet *M*_s_ = 0 CSF. As is familiar in UHF, this additional flexibility lowers the energy and brings the result closer to the exact FCI energy, albeit at the cost of mixing different spin states. The contribution from the triplet CSF decreases smoothly at short distances, but, in contrast to UHF, is still non-zero at bond distances 0.25 Å shorter than *R*_e_: there is no analog of a Coulson–Fischer point, *i.e.*, no collapse to RHF solution. Consequently, the value of 〈*S*^2^〉 deviates from zero (a pure singlet) all along the potential energy curve.

The Re-uCJ ansatz displays an even more intriguing behavior, showing very distinct wavefunction compositions for the two local minima derived from the equilibrium (dissociation ansatz) and separated atom regimes (association ansatz). With the dissociation ansatz (Fig. 3d), the wavefunction derived from equilibrium retains significant overlap with the HF state, increasing the overall energy error at large distances. Additionally, we observe contributions from the open-shell singlet CSF, indicating that while the 〈*S*^2^〉 and 〈*M*_s_〉 are correct for a pure singlet state, the Re-uCJ ansatz exhibits spatial symmetry breaking (the open-shell singlet has ungerade (u) symmetry).

In contrast, the Re-uCJ local minimum derived from the separate atom regime (*i.e.*, the association ansatz, Fig. 3c) behaves quite differently. Initially, in the dissociation regime, the wavefunction contains a mixture of triplet *M*_s_ = 0, doubly excited singlet, and HF singlet CSFs, but as the bond distance decreases, the wavefunction collapses into the RHF solution, rationalizing the energy curve behavior observed in Fig. 1a. Therefore this solution exhibits the analog of a Coulson–Fischer point, and appears to approach 50% triplet character at dissociation, resembling a UHF reference state. Interestingly, its spin contamination exceeds that of the Im-uCJ solution for all distances beyond about 1.2 Å as can be seen from the larger contribution form the triplet *M*_s_ = 0 CSF.

The difference in CSF composition between Re-uCJ, Im-uCJ, and g-uCJ can be rationalized by their different relative expressibility (*i.e.* the flexibility of their functional forms). The Re-uCJ ansatz cannot exactly reproduce the exact ground-state singlet wavefunction (which consists of a combination of Hartree–Fock and doubly excited singlet CSFs). As Fig. 3c shows, Re-uCJ introduces correlation by also introducing symmetry-breaking configurations. Additionally, limitations of the (*k* = 1) Re-uCJ ansatz force higher triplet *vs.* singlet CSF character whenever they are non-zero (giving a lower energy solution only at the dissociation limit). That is why an alternative solution involving the open-shell singlet CSF was located at smaller H–H distances (Fig. 3d). In contrast, the Im-uCJ ansatz accesses a different subspace of the Hilbert space through complex orbital rotations, allowing it to reach the doubly excited singlet CSF through variable inclusion of triplet CSF character at all H–H bond distances (Fig. 3a). In turn, g-uCJ has additional flexibility beyond either variant (Im- or Re-uCJ) individually. Thus, this more expressive circuit recovers the correct composition of CSFs observed in the exact singlet ground state wavefunction (Fig. 3b).

### 3.4 Two-qubit gate costs for unencoded qubit calculations

We estimate here the scaling of the number of gates required for the circuits generating the uCJ ansätze (exact implementation without Trotter decomposition) with bare qubits, *i.e.*, without using any encoding into a quantum error code. In all gate estimates, we assume an all-to-all qubit connectivity in the quantum device, compatible with trapped ion architectures. We assume compilation of the circuits into CNOT gates and arbitrary single qubit rotations *R*_*z*_, where the latter can be further decomposed into order 1.15 × log_2_(1/*ε*_syn_) + 9.2 T gates, with arbitrary synthesis error *ε*_syn_.^89^

The exponential of the *K̂* operator (e^*K̂*^) can be represented as a series of Givens rotations,^73^ which requires 
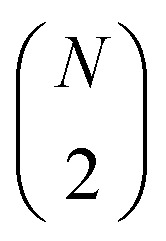
 operations, where *N* is the number of spin–orbitals. Given that our generalized Givens rotation is essentially a two-qubit gate, it can be in general represented with 3 CNOT gates.^74–76^ The exponential of the *Ĵ* operator (e^*Ĵ*^), which consists of paired number operator rotations of the form e^−*iθn̂*_*i*_*n̂*_*j*_^, requires two CNOT gates and one *R*_z_ gate per term. Noting that there are 
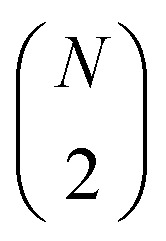
 distinct number operator pair products (without diagonal terms), one can then estimate the maximum number of CNOT gates as:28
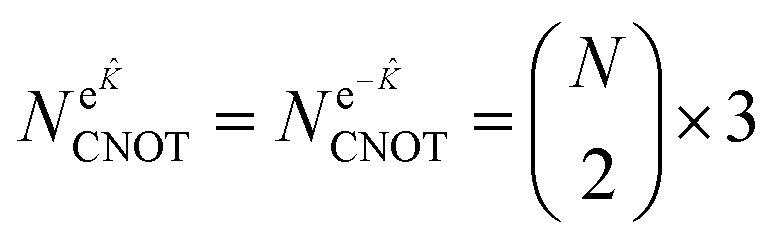
29
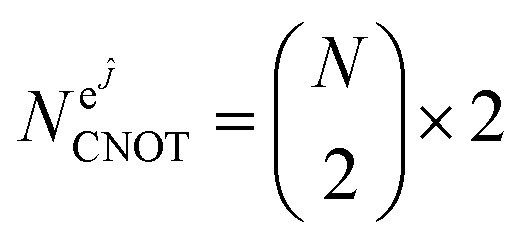


This results in total counts of at most30
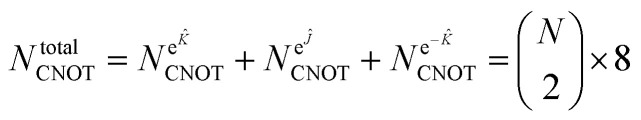
for the circuits to generate individual uCJ ansatz states.

### 3.5 Hardware considerations

In estimating gate counts, we assumed all-to-all qubit connectivity, as is nominally available on ion-trap devices. However, we would like to note one practical limitation that comes with this class of quantum computers. Due to mode crosstalk and spectral crowding, the number of simultaneous entangling operations is limited and requires ion shuttling that introduces a non-negligible time overhead. This makes a maximally parallel implementation of the uCJ circuit challenging. Nevertheless, uCJ is still a viable option for ion-trap devices due to its favorable scalability and expressibility.

Superconducting devices, by contrast, typically allow only nearest-neighbor connectivity but can exploit effective gate parallelization. In cases of limited connectivity, the exact implementation of Givens-rotation operators would still be possible without extra CNOT gate overhead, as it requires only nearest neighbor qubits. The challenge then arises for the e^*Ĵ*^ term, since its decomposition involves factors such as e^*Ẑ*_*i*_*Ẑ*_*j*_^ with distant qubits, which must be realized through chains of CNOT gates. Thus, for superconducting devices, the Local-uCJ variants in ref. 69 provide a natural alternative, by simplifying the Jastrow-factor operator to include only e^*Ẑ*_*i*_*Ẑ*_*j*_^ terms that are connected on a particular device. This effectively reduces expressibility, but this effect can be mitigated by increasing *k*.

We would like to note that, formally, the number of parameters and entangling gates in the *k*-fold uCJ family scales linearly with the parameter *k*. As we noted above, *k* = 1 defines a well-behaved model chemistry that is tractable and is already able to capture a large portion of electron correlation effects. Increasing *k* systematically improves expressibility but comes at the price of a more challenging optimization problem and greater sensitivity to noise. For near-term devices, the recommended strategy is to begin with *k* = 1 and only increase *k* if the uCJ circuit does not achieve the desired accuracy. Overall, we expect ion-trap architectures (with high fidelities and near all-to-all connectivity) to be particularly well suited for uCJ implementations, while superconducting devices may benefit from the more restricted Local-uCJ ansatz with *k* > 1.

### 3.6 Beyond VQE: prognosis for long-term classical optimization of uCJ ansätze and application in NOQE

Looking beyond near-term VQE applications, the uCJ ansätze are naturally compatible with a broader class of hybrid algorithms that decouple the computational steps of parameter optimization and energy evaluation between classical and quantum devices, respectively. In particular, within the framework of the NOQE,^55^ the optimization of the uCJ circuits for various reference states can be performed on classical hardware as was undertaken here in our pre-processing modality. As noted in Section 2.4, this avoids both the measurement shot noise and circuit noise due to hardware constraints, which were seen there to limit the accuracy possible for VQE applications. Another advantage with the NOQE is that the optimization for reference states in the NOQE subspace need not be exact, because of the subsequent subspace diagonalization. Furthermore, while in the current work we have carried out the classical optimization using deterministic methods, the uCJ ansatz is very well suited to optimization by classical Quantum Monte Carlo methods,^67,68^ which is expected to facilitate optimization for larger systems. For significantly larger systems, the fermionic neural network methods in ref. 90–92 constitute an attractive approach for scaling up classical variational optimization of expectation values for multiparameter reference states. The electronic energies can then be refined by the NOQE approach of constructing a generalized eigenvalue problem in a non-orthogonal basis of such classically optimized correlated states, with the requisite matrix elements evaluated on a quantum processor. Thus in NOQE, one would first undertake a classical design and pre-optimization of reference states consisting of non-orthogonal uCJ-dressed states, and follow this by a quantum evaluation of the projected Hamiltonian and overlap matrix elements within the subspace spanned by these reference states. As noted in ref. 55, such a non-orthogonal quantum subspace approach significantly reduces the number of required quantum measurements compared to conventional iterative VQE optimization.

## 4 Conclusions

In this work, we have introduced two new variants of the *k*-fold unitary cluster Jastrow ansatz that are suitable for variational, subspace expansion, or non-orthogonal quantum eigensolver approaches. These are the imaginary unitary cluster Jastrow, Im-uCJ, and the generalized unitary cluster Jastrow, g-uCJ. We evaluated their performance here within a variational framework using single reference states, and choosing the simplest *k* = 1 model. Specifically, we showed that similar to Re-uCJ, it is possible to construct an exact exponentiation procedure for the Im- and g-uCJ ansätze that incorporates generalized orbital rotations, thereby completely avoiding the need for Trotter decomposition.

Our *k* = 1 results show that the restricted Im-uCJ ansatz has the best performance, offering a compelling balance between accuracy and computational efficiency. This ansatz yields shallow circuits, a moderate number of parameters for optimization, and superior performance compared to the previously proposed Re-uCJ ansatz, which often struggles with local minima and convergence issues. The generalized g-uCJ ansatz achieves near-exact accuracy, even reproducing FCI energies for small systems; however it does so at the cost of requiring a larger number of parameters.

A configuration state function (CSF) analysis provided deeper insight into the behavior of these uCJ ansätze. We observed that Im-uCJ lowers the energy at the cost of mixing spin states, while g-uCJ maintains the correct spin symmetry and achieves highly accurate results, albeit with more parameters. On the other hand, Re-uCJ exhibits a strong dependence on initialization, with distinct behaviors in association and dissociation regimes.

We also analyzed the effects on evaluation of energy expectation values in the uCJ ansatz states, of measurement shot noise and circuit noise derived from current hardware limitations on NISQ era machines that do not implement any form of error mitigation or error correction. For single reference states, while the effects of measurement shot noise were not significant, the effects of realistic circuit noise, including both gate and readout errors, were found to limit the accuracy to values outside the desired regime of chemical accuracy. This highlights the limitations of NISQ era hardware for accurate quantum chemical calculations and emphasizes the key importance of hardware developments in the pre-fault tolerant era we are entering now, to enable effective and efficient error mitigation, as well as operation of error-detecting and error-correcting codes. Given the strong performance of the Im-uCJ and g-uCJ ansätze seen in this work in the single reference context with classical pre-optimization, our future objectives are to apply the proposed uCJ circuits within the NOQE algorithm to explore their potential for multi-reference quantum eigensolvers on the pre-fault tolerant quantum hardware currently under development.

In particular, for biradical systems with two broken-symmetry UHF reference states that differ only by a permutation of α and β electrons, the non-orthogonal quantum eigensolver algorithm can improve energy estimates without requiring reoptimization of uCJ parameters. In such cases, only a single parameter set needs to be optimized for Im- or g-uCJ, with the second set readily obtained *via* index permutation in the **K** and **J** matrices. This analysis implies that uCJ ansätze, particularly Im- and g-uCJ, are strong candidates for near-term quantum simulations, offering viable and compact alternatives to more resource-intensive methods like UCCSD. It will also be of interest to apply these alternative unitary cluster Jastrow models to lattice based problems, expanding the capability of the local uCJ ansatz that has been applied recently to Fermi-Hubbard models.^93^

## Author contributions

The article was written with the contributions of all authors.

## Conflicts of interest

There are no conflicts to declare.

## Supplementary Material

SC-016-D5SC03585F-s001

## Data Availability

The data supporting this article have been included as part of the supplementary information (SI)**u**_6_H_6_ (Table S2), Pauli-word groupings for H_2_ STO-3G Hamiltonian (Table S3), and hardware-noise. Any related additional data are available upon request from the authors. Supplementary information: additional figures (S1–S3), discussion of RHF *versus* UHF performance, details of the diagonalization of the matrix **u**, full Cartesian coordinates of all molecular systems (Table S1), CASCI reference energies of C_6_H_6_ (Table S2), Pauli-word groupings for H_2_ STO-3G Hamiltonian (Table S3), and hardware-noise parameters used in noisy simulations (Table S4). See DOI: https://doi.org/10.1039/d5sc03585f.
