# Supplementary material for: Beyond real: alternative unitary cluster Jastrow models for molecular electronic structure calculations on near-term quantum computers
Source: Chem Sci. 2025 Nov 5;16(47):22299–313. doi: 10.1039/d5sc03585f (PMC12628341; doi:10.1039/d5sc03585f)
Supplement: SC-016-D5SC03585F-s001 [file SC-016-D5SC03585F-s001.pdf]

Supplementary Information (SI) for Chemical Science.
This journal is © The Royal Society of Chemistry 2025

Electronic Supplementary Information for the paper entitled:

Beyond real: Alternative unitary cluster Jastrow models for molecular electronic structure calculations on near-term quantum computers

Nikolay V. Tkachenko,[a,b,c,d*] Hang Ren,[a] Wendy M. Billings,[a] Rebecca Tomann,[a] K. Birgitta Whaley[a,e *] and Martin Head-Gordon[a,c,e,*]

[a]*Department of Chemistry, University of California, Berkeley, CA 94720, USA*
[b]*Materials Sciences Division, Lawrence Berkeley National Laboratory, Berkeley, CA 94720, USA*
[c]*Institute for Decarbonization Materials, University of California, Berkeley, CA 94720, USA*
[d]*Department of Chemistry and Biochemistry, University of Oklahoma, Norman, Oklahoma 73019, USA*
[e]*Chemical Sciences Division, Lawrence Berkeley National Laboratory, Berkeley, CA 94720, USA*
E-mail: nikolaytkachenko@berkeley.edu; whaley@berkeley.edu; m_headgordon@berkeley.edu

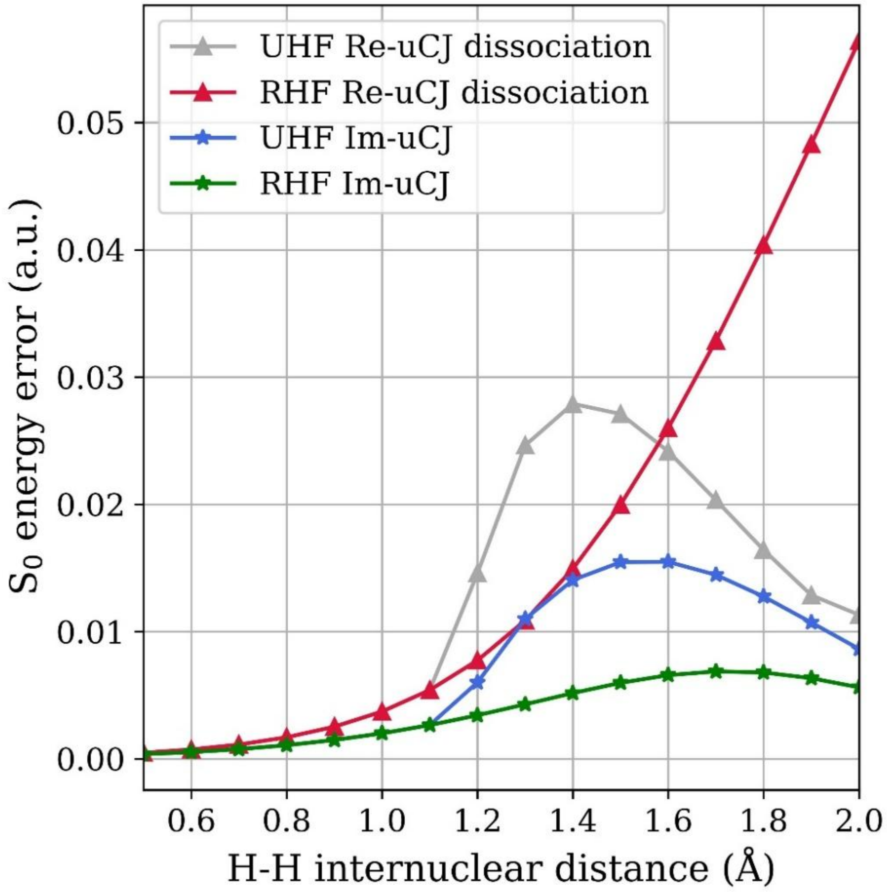

Figure S1. Errors in energies for g-uCJ, re-uCJ and im-uCJ relative to FCI energies for H_2 (STO-3G) with single RHF or UHF reference. The curves denoted by 'dissociation' refer to the procedure of how the initial guesses for the **K** and **J** matrices were obtained. For the dissociation curves, the optimized parameters from the prior step were used as the initial guess at the next step as the bond distance gradually increased. For RHF and UHF Im-uCJ results are independent on the choice of the initial guess for the **K** and **J** matrices. The Coulson-Fischer point is located at H-H internuclear distance of 1.153 Å .

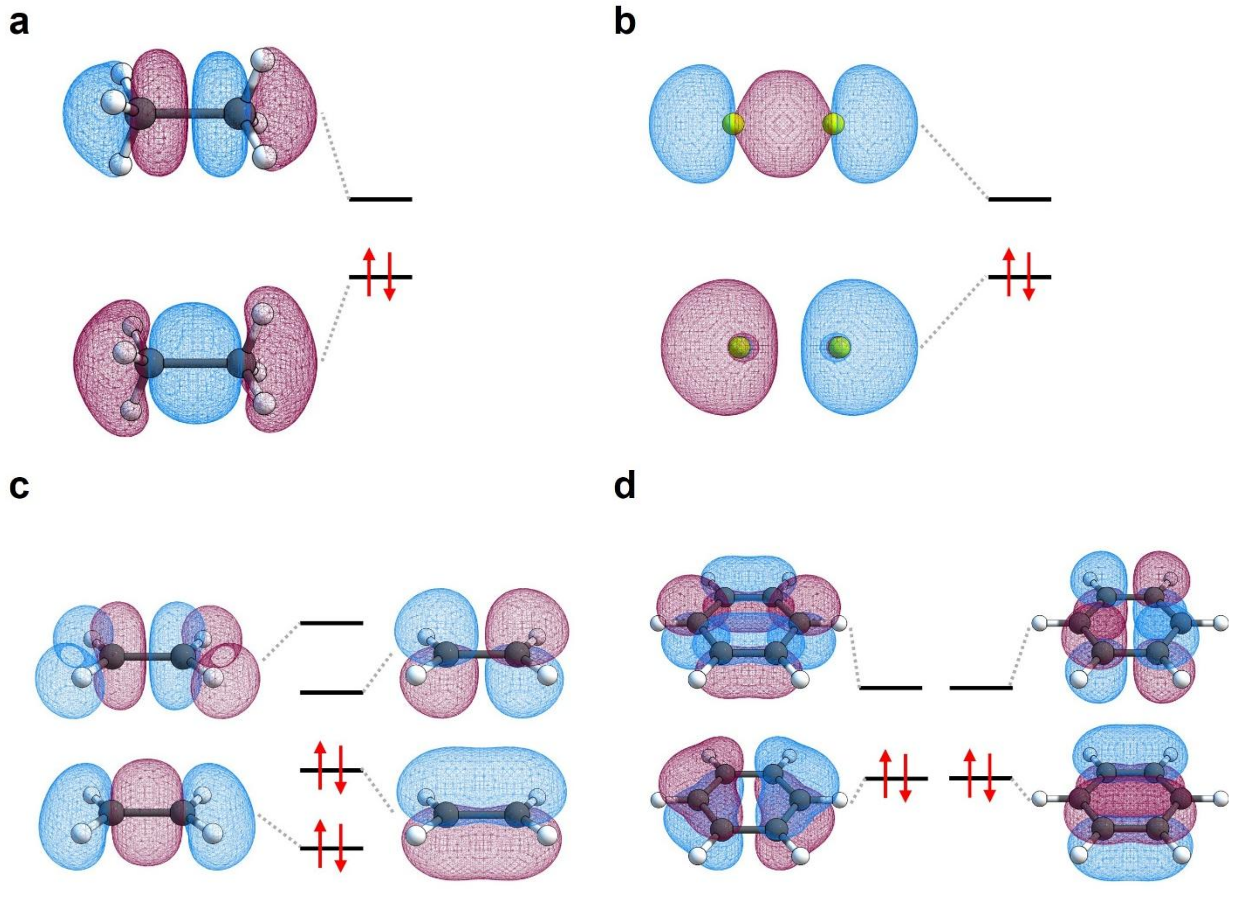

Figure S2. Illustration of active space orbitals used for (a) C_2H_6 (2e,2o), (b) Be_2 (2e,2o), (c) C_2H_4 (4e, 4o), and (d) C_6H_6 (4e, 4o) molecular systems.

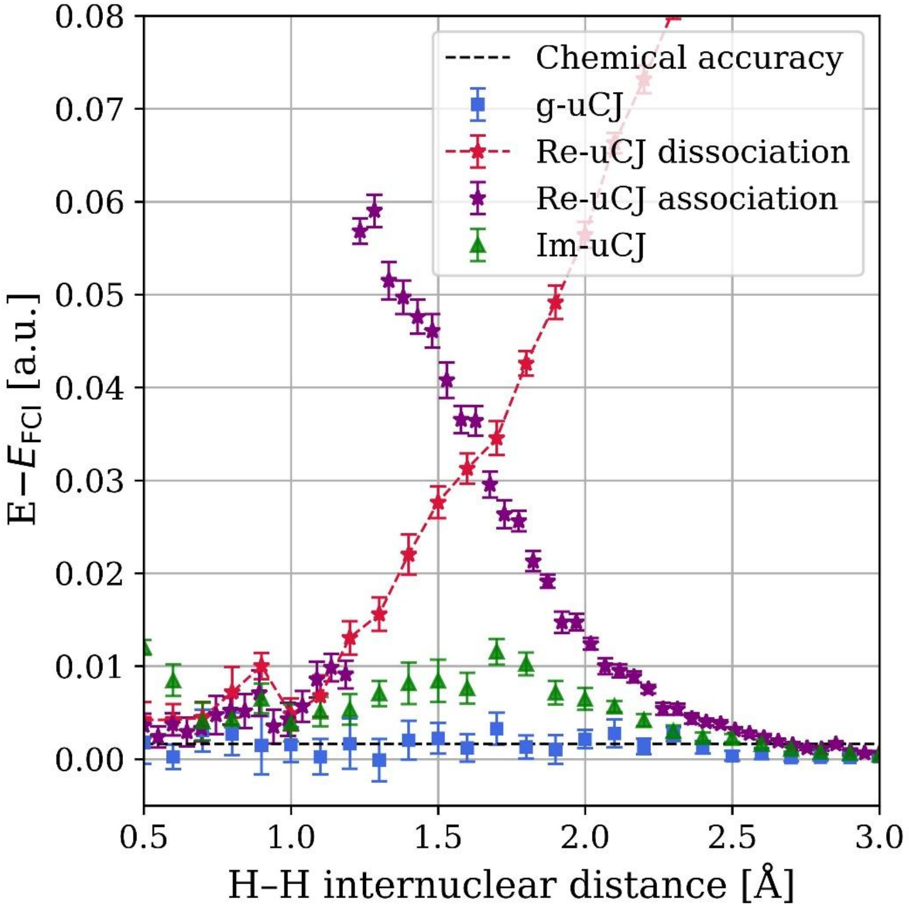

Figure S3. Errors in energies for g-uCJ, re-uCJ and im-uCJ relative to FCI energies for H_2 (STO-3G) with single RHF reference obtained with the QASM-simulator including measurement shot noise. The curves denoted by 'dissociation' refer to the procedure of how the initial guesses for the **K** and **J** matrices were obtained. For the dissociation curves, the optimized parameters from the prior step were used as the initial guess at the next step as the bond distance gradually increased. Error bars are one-sigma standard deviations from mean evaluations.

Discussion of RHF vs UHF performance.

Using the H_2 molecule in the STO-3G basis set as an illustrative example (Figure S1), we clearly observe that beyond the Coulson–Fischer point at internuclear distance 1.153 Å, the RHF reference becomes a more favorable choice than UHF when only a single reference is employed. For the Re-uCJ ansatz, the only potential advantage of using UHF lies in the stability of the optimization. Unlike the RHF-based Re-uCJ, the UHF-based version did not exhibit erratic behavior or multiple local minima during the optimization process. However, this benefit does not extend to the Im-uCJ ansatz, for which such instabilities were not observed with the RHF reference. Therefore, UHF offers no clear advantage in that context. Nevertheless, the broken-symmetry nature of UHF allows for the application of quantum subspace diagonalization (QSD) methods or related methods such as the

NOQE algorithm (ref. 55 of the main text). When used in conjunction with Im-uCJ, this can lead to further improvements in performance, providing justification for the UHF reference in this case. Finally, for the g-uCJ ansatz, both RHF and UHF references yield exact FCI results, demonstrating the robustness of the general uCJ ansatz with respect to the choice of underlying reference state.

Discussion on diagonalization of matrix $\boldsymbol{u}$.

Since the matrix $\boldsymbol{u}$ is generally complex in the Im-uCJ and g-uCJ cases, its elements can be written as

$$u_{ij} = r_{ij} e^{(i\varphi_{ij})} \, ; \; r_{ij} \in \mathbb{R}; \; \varphi_{ij} \in [0,2\pi);$$

Following the description provided in Ref. 68, when the complex Givens rotation matrix $\boldsymbol{r}'_{\boldsymbol{pq}}(\theta,\phi)$, given in Eq. (14), left-multiplies the N by N unitary matrix $\boldsymbol{u}$, the resulting product (matrix $\boldsymbol{u}'$) is also unitary. The modified entries of new matrix $\boldsymbol{u}'$ can be written as:

$$u'_{ij} = \begin{cases} cos(\theta)\, r_{pj} e^{(i\varphi_{pj})} + \sin(\theta)\, r_{qj} e^{\left(i(\pi+\varphi_{qj}-\phi)\right)} & if \;\; i = p \\ \sin(\theta)\, r_{pj} e^{\left(i(\varphi_{pj}+\phi)\right)} + cos(\theta)\, r_{qj} e^{(i\varphi_{qj})} & if \;\; i = q \\ r_{ij} e^{(i\varphi_{ij})} \quad if \; i \neq p,q & \end{cases}$$

To diagonalize $\boldsymbol{u}$ we will use such angles θ and ϕ to make element u'_{qj} equal to zero. Using the expression above we can find that if $\phi = \varphi_{qj} - \varphi_{pj}$ and $\theta = arctan\left(-\frac{r_{qj}}{r_{pj}}\right)$ the element u'_{qj} would be equal to zero.

Table S1. Cartesian coordinates of the molecular systems use in this study.

**Molecule**	**Coordinates**
H_4 square	H 0.000000000 0.000000000 0.000000000<br>H 0.000000000 0.000000000 1.100000000<br>H 0.000000000 1.100000000 0.000000000<br>H 0.000000000 1.100000000 1.100000000
C_2H_6 (dR = uniform grid of 23 points over the interval [–0.2, 2.0])	C 0.000000000 0.000000000 -0.764994000<br>H 1.019690000 0.000000000 -1.164250000<br>H -0.509845000 -0.883078000 -1.164250000<br>H -0.509845000 0.883078000 -1.164250000<br>C 0.000000000 0.000000000 0.764994000 + {dR}<br>H 0.509845000 0.883078000 1.164250000 + {dR}<br>H -1.019690000 0.000000000 1.164250000 + {dR}<br>H 0.509845000 -0.883078000 1.164250000 + {dR}
C_2H_4 (dR = uniform grid of 27 points over the interval [–0.2, 1.0])	C -0.156895360 0.000000000 -0.705755575<br>H -0.156895360 0.923341000 -1.278705575<br>H -0.156895360 -0.923341000 -1.278705575

	C -0.156895360 0.000000000 0.624402425+{dR}<br>H -0.156895360 0.923341000 1.197352425+{dR}<br>H -0.156895360 -0.923341000 1.197352425+{dR}
C_6H_6	C -1.396328944 0.000000000 0.000000000<br>C -0.698164472 -1.209256337 0.000000000<br>C -0.698164472 1.209256337 0.000000000<br>H -1.241338149 -2.150060743 0.000000000<br>H -1.241338149 2.150060743 0.000000000<br>C 0.698164472 -1.209256337 0.000000000<br>C 0.698164472 1.209256337 0.000000000<br>H 1.241338149 -2.150060743 0.000000000<br>H 1.241338149 2.150060743 0.000000000<br>C 1.396328944 0.000000000 0.000000000<br>H 2.482676297 0.000000000 0.000000000<br>H -2.482676297 0.000000000 0.000000000

Table S2. CASCI energies of C_6H_6 obtained with different active spaces. RHF/STO-3G energy of C_6H_6 is -227.890635999114 a.u.

**Active Space**	**Energy**
(4e,4o)	-230.284898601378 a.u.
(6e,6o)	-230.341726882141 a.u.

Table S3. Explicit table of QWC groups for H_2 STO-3G Hamiltonian.

Group basis	Pauli Words
ZZZZ	ZZII, ZIIZ, IZZI, IIZZ, IIIZ, IIZI, IZIZ, ZIZI, IZII, ZIII
YYXX	YYXX
XYYX	XYYX
YXXY	YXXY
XXYY	XXYY

Table S4. Summary of the hardware-noise models used in this work for the H_2 simulations (STO-3G, R(H-H) = 1.7 Å).

Noise model	Native gate set	Error channels included	Representative parameters
**T_1/T_2**	RZ, RX, RZZ	Thermal relaxation + symmetric readout	T_1 = 5 s; T_2 = 1.5 s;<br>1q-gate time = 40 μs;<br>2q-gate time = 300 μs;<br>Readout 0.2 %;
**T_1/T_2 + dep**	RZ, RX, RZZ	Thermal relaxation + depolarizing + symmetric readout	T_1 = 10 s; T_2 = 5 s;<br>1q-gate time = 40 μs;<br>2q-gate time = 300 μs;<br>1q-depolarizing = $2*10^{-6}$;<br>2q-depolarizing = $5*10^{-4}$;<br>Readout 0.1 %;
**H1-like**	RZ, RX, RZZ	Depolarizing + asymmetric readout	1q-depolarizing = $1.8*10^{-5}$;<br>2q-depolarizing = $9.7*10^{-4}$;<br>Readout(0->1) = 0.12%;<br>Readout(1->0) = 0.34%;
**IBM-like**	CZ, RZZ, RX, RZ, SX, X	Thermal relaxation + depolarizing + symmetric readout	T_1 = 304 μs; T_2 = 344 μs;<br>1q-gate time = 32 ns;<br>2q-gate time = 88 ns;<br>1q-depolarizing = $1.687*10^{-4}$;<br>2q-depolarizing = $1.465*10^{-3}$;<br>Readout 0.4456 %;
